# Do Racial/Ethnic and Economic Factors Affect the Rate of Complicated Appendicitis in Children?

**DOI:** 10.1155/2020/3268567

**Published:** 2020-06-29

**Authors:** Abhinav Totapally, Paul Martinez, Andre Raszynski, Fuad Alkhoury, Balagangadhar R. Totapally

**Affiliations:** ^1^Texas Children's Hospital, Baylor College of Medicine, Houston, TX, USA; ^2^Division of Critical Care Medicine, Nicklaus Children's Hospital, Miami, FL 33155, USA; ^3^Herbert Wertheim College of Medicine, Florida International University, Miami, FL 33199, USA; ^4^Department of Pediatric Surgery, Nicklaus Children's Hospital, Miami, FL 33155, USA

## Abstract

**Introduction:**

Appendicitis continues to be one of the most common surgical conditions in the pediatric population. We set out to determine demographic and practice variations among children admitted with appendicitis and highlight the racial/ethnic and healthcare access role in relation to the rate of complicated appendicitis using the 2012 Kids' Inpatient Database (KID). *Methodology*. A retrospective cross-sectional database study was performed using the 2012 KID. All children (age 1 months to 20 years) with appendicitis were identified using the ICD-9 diagnosis codes. Children with a diagnosis of appendicitis were compared with all other discharges. Among children with appendicitis, demographic and practice variations and the rate of complicated appendicitis were evaluated. Univariate and multivariate analyses were done to analyze the data. Sample weighing was done to present national estimates.

**Results:**

In 2012, a total of 89, 935 out of 2.7 million pediatric hospital discharges (3.3%) had a diagnosis of appendicitis. The incidence of appendicitis was higher in males (4.7%), 6–15-year age group (7.43%), Hispanics (5.2%), and in the Western region (5.2%) and was lower in infants (0.02%) and African American children (1.2%) (*p* < 0.0001). The proportion of children with peritonitis or abscess was higher in children's hospitals (48.2% vs. 29.0%; OR 2.3, 95% CI: 2.2–2.4). The risk of complicated appendicitis was inversely related to age, while racial and ethnic minority status, bottom quartile of the income group, and government insurance increased the risk. Laparoscopic appendectomy was performed more frequently at children's hospitals (84.8% vs. 74.3%; *p* < 0.0001).

**Conclusions:**

Appendicitis is more common in Hispanics, males, older children, and in the Western region. Complicated appendicitis is more common in younger children, minority groups, low-income group, and children with government insurance. Children's hospitals manage more children with complicated appendicitis and are more likely to perform laparoscopic appendectomy.

## 1. Introduction

Appendicitis is a common surgical condition with a highest incidence of 23.3 per 10,000 population per year in the 10–19-year age group [1]. The incidence of appendicitis has decreased over the years [1, 2]. There are age, gender, racial, geographic, and seasonal differences in the incidence of appendicitis [1]. The appendicitis rate is highest among teens and males, and it occurs most frequently during summer months [1, 3]. In children, complicated appendicitis is relatively common, and the rate of perforated appendicitis varies with age, the presence of obesity, socioeconomic status, and healthcare access [4–9]. Appendicitis may be managed with an open or laparoscopic appendectomy or with medical management and delayed appendectomy. A study from the PHIS database demonstrated an increased trend for laparoscopic appendectomy in children's hospitals [10]. The epidemiology, complications, and treatment of acute appendicitis continue to change [11]. Using the Healthcare Cost and Utilization Project's (HCUP) 2012 Kids' Inpatient Database (2012 KID), this study explores the recent epidemiology of appendicitis and its management and outcomes in children in the U.S and looks at the factors affecting the risk of complicated appendicitis.

## 2. Methods

### 2.1. Data Source

The Agency for Healthcare Research and Quality and HCUP data use agreement and training which were completed prior to the KID data set analysis. The research proposal was reviewed by the institutional review board and was deemed exempt and classified as nonhuman subject research. We performed a retrospective analysis of the 2012 KID database to determine demographic and practice variations among children admitted with appendicitis. We compared those variations between children's and non-children's hospitals and examined the variable that may influence the diagnosis of complicated appendicitis. Neonates were excluded from the analysis. Duplicated records were excluded to ensure that we did not count the same patient twice.

### 2.2. Patient Selection

Children with a diagnosis of appendicitis were extracted from the database, and demographic variables such as gender, race, age groups, income quartiles, region, and urban vs. rural locations were compared with the rest of the discharges. We further subdivided children with appendicitis into four groups: appendicitis with peritonitis, with abscess, without peritonitis, and unspecified using ICD-9 codes 540.0, 540.1, 540.9, and 541, respectively. Additionally, we compared surgical management of appendicitis which included laparoscopic appendectomy, nonlaparoscopic appendectomy, and no appendectomy. ICD-9 procedure codes (47.0, 47.01, and 47.09) were used to extract different surgeries. Those patients who were transferred to another acute care hospital were excluded to avoid double counting. Finally, we examined the incidence, demographics, and surgical practice variations between children and non-children hospitals amongst appendicitis patients.

### 2.3. Statistical Analysis

We have used chi-square test to analyze categorical variables and multi-chi-square tests for variables with more than two subgroups, such as age groups, race, region, and income. Age-specific trend analysis for incidence of appendicitis, type of surgery, and perforation rate was performed using chi-square trend analysis using Epi Info (CDC, Atlanta, GA).

Patients were grouped into 5 age groups: infants (less than 1 year); 1–5 years; 6–10 years; 11–15 years; and 16–20 years. HCUP defines median household income (MHI) by the ZIP code in which the child resides. The ZIP codes are stratified by income quartiles with quartile 1 representing the lowest and quartile 4 representing the highest income. Appendicitis with perforation or with abscess was classified as complicated appendicitis, and the remainder were classified as noncomplicated appendicitis. The proportion of complicated appendicitis out of the total number of children with appendicitis discharged from the hospitals was compared among various demographic variables. Race and ethnicity were grouped into White, African American, Hispanic, and others (Asian, Native American, and unknown). Payer status was grouped into government insurance (Medicare and Medicaid), private insurance, and others. Hospital regions were divided into Northeast, Midwest, South, and West. The data were weighted to give national estimates. Multivariate analysis was conducted to determine the adjusted risk of complicated appendicitis.

## 3. Results

In 2012, a total of 89, 935 out of 2.7 million pediatric hospital discharges (3.3%) had a diagnosis of appendicitis. Nineteen patients died with a mortality rate of 0.021%. Within the appendicitis group, 20.6% had peritonitis, 12% had a peritoneal abscess, 64.7% had no peritonitis, and 3.4% of cases were unspecified.

### 3.1. Demographics

Males comprised 59.4% of all appendicitis patients. The incidence of appendicitis was higher among males (4.65 vs. 2.37 per 100 patients; RR 1.43, 95% CI: 1.41–1.44). Infants had the lowest rates of appendicitis (0.1%), while patients in the 6–10-year and 11–15-year groups had the highest incidence (7.72% and 7.42%, respectively; *p* < 0.0001). The incidence was highest in Hispanic patients at 5.19 per 100 patients discharged and lowest in African American patients at 1.18 per 100 patients discharged (*p* < 0.001). Obesity was present in 3.3% (95% CI: 3.1–3.3%) of children discharged with a diagnosis of appendicitis. There was a significant seasonal variation in the incidence of appendicitis with the highest rate in summer months and the lowest rate in winter months (*p* < 0.0001). The incidence of appendicitis was 2.75% in the lowest quartile MHI and 4.31% in the highest quartile MHI (*p* < 0.0001). The incidence of appendicitis in urban areas was significantly higher than that in rural areas (3.38% vs. 2.88%; RR: 1.17, 95% CI 1.45–1.2, *p* < 0.001). There was a significant regional variation in the incidence of appendicitis with the highest incidence of appendicitis in the West at 5.15% and the lowest in the Midwest at 2.51% (*p* < 0.0001). Severe sepsis or septic shock was present in 0.3% of appendicitis patients, while 0.2% of children required mechanical ventilation.  Table 1 shows demographic details.

Of all cases of appendicitis, 7.28% (*n* = 6,514) were transferred-in from another acute care hospital. Of the transfer-in cases, 27.68% and 16.89% had peritonitis and abscesses, respectively. This is significantly greater than in the nontransfer patients (*p* < 0.0001). An almost equal proportion of both transfer-in and nontransfer cases was managed surgically with an appendectomy (94.41 vs. 90.01%). As expected, the proportion of transfer-in cases was higher in children's hospitals than compared with non-children's hospitals (25.40% vs. 15.65%; *p* < 0.0001).

### 3.2. Complicated Appendicitis

**Figure 1:**
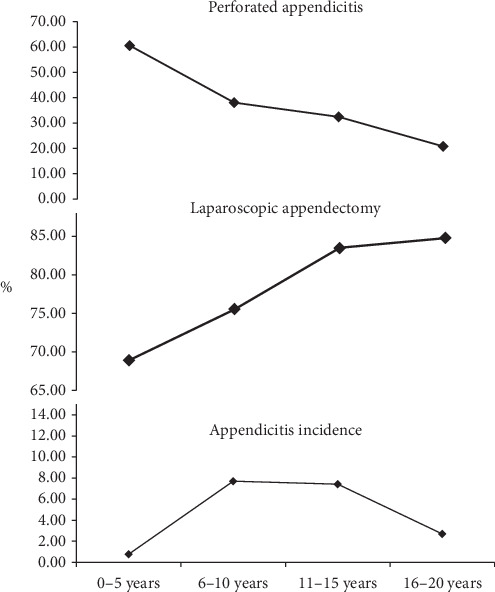
Age-specific trends in incidence of appendicitis, appendicitis with peritonitis or abscess, and laparoscopic appendectomy (trend analysis; *p* < 0.0001).

Overall, complicated appendicitis was present in 32.4% of all discharges with appendicitis. On univariate analysis, the proportion of complicated appendicitis was highest in the 1–5-year age group at 61.3% and lowest in the 16–20-year age group at 20.9% (*p* < 0.0001; Figure 1). The proportion of children with complicated appendicitis was lowest in the Northeast (25.7%) and highest in the Midwest (37.7%) among the four regions of the United States (*p* < 0.0001). The complicated appendicitis rate was lowest in White children (29.7%) and highest in the other race/ethnicity groups (36%; *p* < 0.0001). The incidence of complicated appendicitis varied with insurance status. It was lowest in children with private insurance (29.6%) and highest in children with government insurance (36.2%; *p* < 0.0001). The incidence of complicated appendicitis was lowest (28.6%) in children living in ZIP code areas with 4th quartile of median household income (*p* < 0.0001). Complicated appendicitis was more common in males compared with females (33.1% vs. 31.3%; RR: 1.05, 95% CI: 1.03–1.07; *p* < 0.0001). Children who were discharged from children's hospitals with a diagnosis of appendicitis were more likely to have complicated appendicitis (48.3% vs. 29.3%; RR: 1.16, 95% CI: 1.15–1.18, *p* < 0.0001).  Table 2 describes the incidence of complicated appendicitis based on various demographic characteristics.  Table 3 demonstrates the adjusted risk for complicated appendicitis based on a multivariate analysis. African American, Hispanic, and other racial/ethnic groups compared with White children and younger children (0–5 years) were at higher risk for complicated appendicitis. Having private insurance compared with government insurance, living in ZIP codes with median household income in the 4th quartile compared with the 1st quartile, was associated with a lower risk of complicated appendicitis. All other risk factors are presented in Table 3.

### 3.3. Management of Appendicitis

Surgical management included laparoscopic appendectomy in 76% of cases, nonlaparoscopic appendectomy in 18.1% of cases, and no appendectomy in 5.9% of cases. Characteristics of children with various surgical approaches are presented in Table 4. Only 1,683 (1.9%) patients had documentation of antibiotic treatment.

### 3.4. Children's Hospitals vs. Non-Children's Hospitals

Only 16.4% of appendicitis cases were managed at a children's hospital, while 83.6% were managed at non-children's hospitals. The incidence of appendicitis diagnosed at children's hospitals was significantly less than the incidence at non-children's hospitals (2.81% vs. 3.47%; RR: 0.994, 95% CI: 0.993–0.994, *p* < 0.0001). Approximately 32.05% and 16.15% of patients with appendicitis at children's hospitals had peritonitis and abscesses (48.2% complicated appendicitis), respectively, compared with only 18.07% and 10.93% (29% complicated appendicitis), respectively, of non-children's hospital patients (*p* < 0.05). The most common surgical course for both children's and non-children's hospitals was laparoscopic appendectomy, but laparoscopic surgery was more common at children's hospitals (84.79% vs. 74.33%; *p* < 0.0001). Nonlaparoscopic/open appendectomy was less common (5.79% vs. 20.44%), and nonsurgical management was more common (9.43% vs. 5.23%) in children's hospitals (*p* < 0.0001).

### 3.5. Age-Specific Incidence and Complications

As stated earlier, the incidence of appendicitis peaks between ages 6 and 15 years. However, the incidence of complicated appendicitis was highest amongst children aged 0–5 years at 60.6%. The incidence of complicated appendicitis was inversely related to age. In terms of surgical management, the proportion of laparoscopic appendectomy was directly related to age with ages 16–20 years demonstrating highest incidence of 81% (*p* < 0.0001; Figure 1).

### 3.6. Mortality

The overall mortality rate in children with appendicitis was 0.02% which was lower than the mortality rate in all other discharges (0.31%; RR: 0.969, 95% CI: 0.968–0.970, *p* < 0.0001).

## 4. Discussion

Our study shows significant regional, seasonal, and age-group variations in the incidence and complications of appendicitis. Racial/ethnic minorities, children in the low-income group, and children with government insurance have higher risk of complicated appendicitis. Younger children have a lower incidence of appendicitis, but they present with complicated appendicitis more often. Laparoscopic appendectomy was performed more often in children's hospitals and in older children.

The incidence of appendicitis was found to be higher in high-income quartiles and in children with private insurance. In contrast, in a study from Taiwan, the incidence of appendicitis and perforation was higher among low-income population [12]. Racial and ethnic variation in the incidence and complications in our study is similar to a previous study from the KID database [13]. The incidence of appendicitis is lower in African American children compared with Whites and higher in Hispanics compared with Whites. As with our study, the prevalence of complex appendicitis was higher in African Americans and Hispanics compared with White children in a previous study [13].

Higher incidence of appendicitis during summer in our study was similar to that seen in Ontario, Canada [14], in California [15], in the US [1], in Iran [16], in Italy [3], in Nigeria [17], and in Taiwan [18]. Although appendicitis is most common in summer months, rates of perforated appendicitis are highest in fall and winter [8]. The reason for seasonal variation with highest incidence in the summer and the lowest in winter is not known. A possibility of more exposure to infectious agents and allergens during summer months has been suggested [17].

The higher prevalence of complicated appendicitis in younger children as demonstrated in our study has been described previously [9]. The incidence of ruptured appendicitis may be used as a quality indicator for access to care [9]. In the present study, complicated appendicitis was more frequent in lower-income quartiles and in children with government insurance compared with private insurance. Racial/ethnic disparities in access to care may be related to higher prevalence of complicated appendicitis in African American and Hispanic children and other minority groups. Ruptured appendicitis rates were more frequent in African Americans compared with Whites in high-impact areas after Hurricane Katrina, which may be related to racial healthcare disparities [19]. In a study from California and New York, it was found that the odds of ruptured appendicitis was increased by as much as 47% for African American children, 45% for Hispanic children, and 116% for Asian American children compared with that for White children [20]. In our study, nationally, similar findings were observed. Ruptured appendicitis is more common in rural Ohio where the distance to the acute care hospital was greater [21]. Even with universal healthcare, rural and low socioeconomic status are associated with a higher incidence of ruptured appendicitis [6]. Increasing geographic density of pediatricians is associated with a decreasing trend in the odds ratio of perforated appendicitis [7]. Rural/urban gap in the risk of complicated appendicitis was not observed in our national study. The racial disparities in the incidence of ruptured appendicitis may be more related to access to care and timely referral than disparities in care once the child reaches a hospital [22]. The rate of ruptured appendicitis has been suggested as an indicator for healthcare access [23]. The knowledge of regional, economic, and racial/ethnic variations in the incidence of complicated appendicitis is important with respect to public health preventive measures such as improved healthcare access and preventive care visits.

Although laparoscopic approach has been used in all ages and all stages of appendicitis (noncomplicated, perforated, and appendicitis with abscess), it took longer time for laparoscopic procedure in children compared to the open approach [24]. Laparoscopic appendectomy is at least as safe and effective as, if not superior to, open approach for both simple and perforated appendicitis [25]. Postoperative pain is less, and recovery is faster with laparoscopic approach, thereby reducing LOS and overall cost [25]. Laparoscopic appendectomy has become a procedure of choice in children [25]. Laparoscopic appendectomy was the most common approach used in our study, across age groups, and locations. The use of laparoscopic approach for appendectomy in children has significantly increased in children's hospitals [10]. However, laparoscopic approach was less often used in younger children, in children with complicated appendicitis, and in non-children's hospitals. Surgeon experience of laparoscopic approach in children may account for the variation between children's and non-children's hospitals.

There are several limitations to our study. Due to the retrospective nature and lack of granularity of data collection in an administrative database, we were not able to evaluate specific details of the surgical procedure, antibiotic use, and so on. Because of the cross-sectional nature of the KID database, we were unable to determine long-term complications and readmissions. The extent of coding errors and under-reporting of the prevalence cannot be determined.

## 5. Conclusions

In summary, this retrospective analysis of a large national patient sample describes the epidemiology, regional, seasonal, and racial/ethnic variations in the incidence, complications, and management of acute appendicitis in the US. Acute appendicitis is common in Hispanic children, in older children and early teens, in the Western US region, in the high-income quartile, and during summer months. Complicated appendicitis is more common in racial/ethnic minorities, low-income groups, children with public insurance, and those treated in children's hospital. The knowledge of variation in complicated acute appendicitis across the geography and demographics will help in public health planning.

## Figures and Tables

**Table 1:** Demographic characteristics of children discharged with a diagnosis of appendicitis and no appendicitis during 2012 in the United States.

Category	Appendicitis		Nonappendicitis		Incidence of appendicitis	Significance
	*N* (95% CI)	Proportion	*N* (95% CI)	Proportion		
Gender						
Male	53,459 (52,926–53,991)	59.4% (59.1%–59.8%)	1,117,099 (1,115,221–1,118,976)	42.3% (42.2%–42.4%)	4.6% (4.5%–4.6%)	RR: 1.955 (95% CI: 1.925–1.985)
Female	36,475 (36,033–36,917)	40.6% (40.2%–40.9%)	1,524,697 (1,522,796–1,526,598)	57.7% (57.6%–57.8%)	2.3% (2.3%–2.4%)	

Age groups						
0-1 year	53 (36–70)	0.06% (0.04%–0.08%)	335,621 (334,355–336,887)	12.70% (12.7%–12.8%)	0.02% (0.01%–0.02%)	*p* < 0.0001
1–5 years	6,818 (6,626–7,010)	7.6% (7.4%–7.8%)	543,579 (542,050–545,107)	20.6% (20.5%–20.6%)	1.2% (1.2%–1.3%)	
6–10 years	23,258 (22,904–23,612)	25.9% (25.5%–26.2%)	282,554 (281,384–283,725)	10.7% (10.7%–10.7%)	7.6% (7.5%–7.7%)	
11–15 years	29,800 (29,400–30,200)	33.1% (32.8%–33.5%)	378,557 (377,230–379,884)	14.3% (14.3%–14.4%)	7.3% (7.2%–7.4%)	
16–20 years	30,006 (29,607–30,406)	33.4% (33.0%–33.7%)	1,101,777 (1,099,997–1,103,557)	41.7% (41.6%–41.8%)	2.7% (2.6%–2.7%)	

Race/ethnicity						
White	42,743 (42,264–43,222)	47.5% (47.1%–47.9%)	1,208,008 (1,206,122–1,209,893)	45.7% (45.7%–45.8%)	3.4% (3.4%–3.5%)	*p* < 0.0001
Black	5,691 (5,514–5,867)	6.3% (6.1%–6.5%)	481,980 (480,532–483,427)	18.2% (18.2%–18.3%)	1.2% (1.1%–1.2%)	
Hispanic	28,944 (28,554–29,335)	32.2% (31.8%–32.5%)	536,019 (534,557–537,482)	20.3% (20.2%–20.3%)	5.1% (5.1%–5.2%)	
Others	12,557 (12,297–12,817)	14.0% (13.7%–14.2%)	416,081 (414,723–417,440)	15.7% (15.7%–15.8%)	2.9% (2.9%–3.0%)	

Seasons						
Spring	23,321 (22,967–23,675)	25.9% (25.6%–26.3%)	671,637 (669,973–673,301)	25.4% (25.4%–25.5%)	3.4% (3.3%–3.4%)	*p* < 0.001
Summer	23,476 (23,121–23,831)	26.1% (25.8%–26.4%)	612,360 (610,751–613,970)	23.2% (23.1%–23.2%)	3.7% (3.6%–3.7%)	
Fall	21,174 (20,836–21,511)	23.5% (23.2%–23.9%)	648,793 (647,146–650,440)	24.6% (24.5%–24.6%)	3.2% (3.1%–3.2%)	
Winter	21,964 (21,621–22,308)	24.4% (24.1%–24.8%)	709,298 (707,599–710,997)	26.8% (26.8%–26.9%)	3.0% (3.0%–3.1%)	

Regions						
Northeast	16,628 (16,334–16,922)	18.5% (18.2%–18.8%)	467,401 (466,752–468,050)	17.7% (17.7%–17.7%)	3.4% (3.4%–3.5%)	*p* < 0.0001
Midwest	14,965 (14,689–15,241)	16.6% (16.4%–16.9%)	591,402 (590,595–592,208)	22.4% (22.4%–22.4%)	2.5% (2.4%–2.5%)	
South	28,961 (28,559–29,363)	32.2% (31.8%–32.6%)	1,032,818 (1,031,924–1,033,712)	39.1% (39.1%–39.1%)	2.7% (2.7%–2.8%)	
West	29,382 (28,999–29,765)	32.7% (32.3%–33.0%)	550,468 (549,621–551,314)	20.8% (20.8%–20.9%)	5.1% (5.0%–5.1%)	

Median household income for patient ZIP code						
1st quartile	24,834 (24,465–25,202)	28.2% (27.8%–28.5%)	890,762 (889,010–892,515)	34.5% (34.4%–34.6%)	2.7% (2.7%–2.8%)	*p* < 0.0001
2nd quartile	21,153 (20,815–21,492)	24.0% (23.7%–24.3%)	647,040 (645,411–648,670)	25.0% (25.0%–25.1%)	3.2% (3.1%–3.2%)	
3rd quartile	21,297 (20,961–21,634)	24.2% (23.8%–24.5%)	575,423 (573,870–576,975)	22.3% (22.2%–22.3%)	3.6% (3.5%–3.6%)	
4th quartile	20,818 (20,488–21,148)	23.6% (23.3%–24.0%)	469,783 (468,373–471,193)	18.2% (18.1%–18.2%)	4.2% (4.2%–4.3%)	

Insurance						
Government	36,422 (35,979–36,864)	40.5% (40.1%–40.9%)	1,416,366 (1,414,447–1,418,285)	53.6% (53.5%–53.7%)	2.5% (2.5%–2.5%)	*p* < 0.0001
Private	43,883 (43,401–44,364)	48.8% (48.4%–49.2%)	994,609 (992,769–996,450)	37.6% (37.6%–37.7%)	4.2% (4.2%–4.3%)	
Others	9,631 (9,402–9,860)	10.7% (10.5%–11.0%)	231,113 (230,036–232,190)	8.7% (8.7%–8.8%)	4.0% (3.9%–4.1%)	

Urban vs. rural						
Urban	76,543 (75,914–77,172)	85.1% (84.8%–85.4%)	2,190,504 (2,189,244–2,191,764)	82.9% (82.9%–83.0%)	3.4% (3.3%–3.4%)	RR: 1.172 (95% CI: 1.147–1.198)
Rural	13,392 (13,116–13,668)	14.9% (14.6%–15.2%)	451,584 (450,456–452,712)	17.1% (17.0%–17.1%)	2.9% (2.8%–2.9%)	

Hospital type						
Children's hospital	14,724 (14,438–15,010)	16.4% (16.1%–16.7%)	514,364 (514,042–514,686)	19.5% (19.5%–19.5%)	2.8% (2.7%–2.8%)	RR: 0.994 (95% CI: 0.993–0.994)
Non-children's hospital	75,212 (74,589–75,834)	83.6% (83.3%–83.9%)	2,127,724 (2,127,088–2,128,359)	80.5% (80.5%–80.5%)	3.4% (3.4%–3.4%)	

**Table 2:** Characteristics of children with complicated appendicitis and noncomplicated appendicitis discharged during 2012 in the United States.

Category	Complicated appendicitis^*∗*^		Noncomplicated appendicitis		Significance
	*N* (95% CI)	Proportion	*N* (95% CI)	Proportion	
Gender					
Male	17,726 (17,450–18,002)	60.8% (60.2%–61.5%)	35,733 (35,393–36,073)	58.8% (58.3%–59.2%)	RR: 1.052 (95% CI: 1.031–1.074); *p* < 0.0001
Female	11,413 (11,182–11,645)	39.2% (38.5%–39.8%)	25,062 (24,751–25,373)	41.2% (40.8%–41.7%)	

Age groups					
0-1 year	18 (8–28)	0.1% (0.0%–0.1%)	35 (21–48)	0.1% (0.0%–0.1%)	*p* < 0.0001
1–5 years^*∗∗*^	4,183 (4,037–4,328)	14.4% (13.9%–14.8%)	2,636 (2,518–2,753)	4.3% (4.1%–4.5%)	
6–10 years^*∗∗*^	8,931 (8,723–9,138)	30.6% (30.0%–31.3%)	14,327 (14,073–14,582)	23.6% (23.2%–24.0%)	
11–15 years	9,731 (9,514–9,947)	33.4% (32.7%–34.0%)	20,069 (19,779–20,360)	33.0% (32.6%–33.5%)	
16–20 years^*∗∗*^	6,279 (6,102–6,456)	21.5% (21.0%–22.1%)	23,728 (23,432–24,024)	39.0% (38.6%–39.5%)	

Race/ethnicity					
White^*∗∗*^	12,685 (12,443–12,927)	43.5% (42.9%–44.2%)	30,058 (29,739–30,377)	49.4% (49.0%–49.9%)	*p* < 0.0001
Black	1,839 (1,739–1,938)	6.3% (6.0%–6.7%)	3,852 (3,711–3,993)	6.3% (6.1%–6.6%)	
Hispanic^*∗∗*^	10,097 (9,882–10,312)	34.6% (34.0%–35.3%)	18,848 (18,576–19,119)	31.0% (30.6%–31.4%)	
Others^*∗∗*^	4,520 (4,368–4,672)	15.5% (15.0%–16.0%)	8,037 (7,841–8,234)	13.2% (12.9%–13.5%)	

Regions					
Northeast^*∗∗*^	4,267 (4,134–4,401)	14.6% (14.2%–15.1%)	12,360 (12,221–12,499)	20.3% (20.1%–20.5%)	*p* < 0.0001
Midwest^*∗∗*^	5,639 (5,493–5,785)	19.3% (18.9%–19.8%)	9,326 (9,182–9,470)	15.3% (15.1%–15.6%)	
South^*∗∗*^	9,792 (9,591–9,993)	33.6% (33.0%–34.2%)	19,169 (18,971–19,368)	31.5% (31.3%–31.8%)	
West	9,443 (9,251–9,635)	32.4% (31.8%–33.0%)	19,938 (19,741–20,136)	32.8% (32.5%–33.1%)	

Median household income for the patient ZIP code					
1st quartile^*∗∗*^	8,430 (8,226–8,633)	29.5% (28.9%–30.1%)	16,404 (16,143–16,666)	27.6% (27.2%–28.0%)	*p* < 0.0001
2nd quartile^*∗∗*^	7,208 (7,019–7,397)	25.2% (24.6%–25.8%)	13,945 (13,694–14,197)	23.4% (23.0%–23.8%)	
3rd quartile	6,985 (6,799–7,170)	24.4% (23.9%–25.0%)	14,313 (14,061–14,564)	24.0% (23.6%–24.5%)	
4th quartile^*∗∗*^	5,961 (5,790–6,132)	20.9% (20.3%–21.4%)	14,857 (14,609–15,104)	25.0% (24.6%–25.4%)	

Insurance					
Government^*∗∗*^	13,171 (12,926–13,415)	45.2% (44.5%–45.9%)	23,251 (22,947–23,555)	38.2% (37.8%–38.7%)	<0.01
Private^*∗∗*^	13,009 (12,766–13,253)	44.6% (44.0%–45.3%)	30,873 (30,548–31,199)	50.8% (50.3%–51.2%)	
Others^*∗∗*^	2,961 (2,835–3,086)	10.2% (9.8%–10.6%)	6,670 (6,488–6,853)	11.0% (10.7%–11.3%)	

Hospital type					
Children's hospital	7,108 (6,963–7,253)	24.4% (23.9%–24.8%)	7,616 (7,470–7,761)	12.5% (12.3%–12.7%)	RR: 1.16 (95% CI: 1.15–1.17); *p* < 0.0001
Non-children's hospital	22,033 (21,747–22,319)	75.6% (75.2%–76.1%)	53,179 (52,893–53,465)	87.5% (87.3%–87.7%)	

Others					
Length of stay (days)^*∗∗∗*^	5.25 (5.19–5.31)		1.77 (1.75–1.80)		Mean difference: 3.49 (95% CI: 3.44–3.54); *p* < 0.0001
Charges ($)^*∗∗∗*^	44,316 (43,697–44,934)		28,813 (28,473–29,152)		Mean difference: 15,503 (95% CI: 14,906–16,100); *p* < 0.0001

^*∗*^Complicated appendicitis = appendicitis with perforation or abscess. ^*∗∗*^Groups significantly different in multi-chi-square analyses. ^*∗∗∗*^Presented as mean (95% CI).

**Table 3:** Multivariable analysis of risk factors for complicated appendicitis.

Potential risk factors (model *N* = 62,756)	Adjusted odds ratio for complicated appendicitis	95% confidence interval		*p* value^*∗*^
Age groups (ref = 0–5 years)				—
6–10 years	0.42	0.39	0.44	<0.001
11–15 years	0.34	0.32	0.36	<0.001
16–20 years	0.21	0.19	0.22	<0.001
Race/ethnicity (ref = White)				—
African American	1.08	1.02	1.15	0.02
Hispanic	1.07	1.03	1.11	<0.001
Others	1.17	1.12	1.23	<0.001
Female (ref = male)	0.92	0.89	0.95	<0.001
Household income quartiles (ref = 1st quartile)				
2nd quartile	1.02	0.98	1.06	0.33
3rd quartile	0.99	0.95	1.04	0.79
4th quartile	0.90	0.86	0.95	<0.001
Transfer-in (ref = not a transfer)				
Transferred from another acute care hospital	1.39	1.32	1.47	<0.001
Transferred in from other types of facility	1.00	0.90	1.12	0.94
Hospital region (ref = Northeast)				—
Midwest	1.58	1.50	1.67	<0.001
South	1.31	1.25	1.37	<0.001
West	1.15	1.10	1.20	<0.001
Urban/rural (ref = urban)	0.99	0.94	1.03	0.61
Season (ref = summer)				—
Spring	1.10	1.06	1.14	<0.001
Fall	1.09	1.04	1.14	<0.001
Winter	1.08	1.04	1.13	<0.001
Insurance (ref = Govt insurance)				
Private insurance	0.94	0.91	0.97	<0.001
Others	1.02	0.97	1.08	0.35
Hospital type (ref = non-children's hospital)	1.70	1.64	1.77	<0.001

^*∗*^ *p* values calculated from Binary regression.

**Table 4:** Characteristics of children with appendicitis operated laparoscopically or through open approach during 2012 in the United States.

Category	Laparoscopic appendectomy		Open appendectomy		No appendectomy		Significance
	*N* (95% CI)	Proportion	*N* (95% CI)	Proportion	*N* (95% CI)	Proportion	
Gender							
Male	40,404 (40,059–40,750)	75.6% (75.1%–76.0%)	10,044 (9,827–10,261)	18.8% (18.4%–19.2%)	3,011 (2,886–3,136)	5.6% (5.4%–5.9%)	*p* < 0.001
Female	27,985 (27,662–28,307)	76.7% (76.2%–77.2%)	6,183 (6,008–6,359)	17.0% (16.5%–17.4%)	2,307 (2,197–2,417)	6.3% (6.0%–6.6%)	

Age groups							
0-1 year	19 (8–29)	35.1% (21.6%–51.4%)	22 (11–33)	41.8% (27.4%–57.8%)	12 (4–20)	23.1% (12.4%–38.8%)	*p* < 0.003
1–5 years	4,146 (4,002–4,290)	60.8% (59.4%–62.2%)	1,858 (1,759–1,957)	27.2% (26.0%–28.5%)	815 (749–881)	11.9% (11.1%–12.9%)	
6–10 years	16,451 (16,186–16,715)	70.7% (70.0%–71.4%)	5,336 (5,172–5,500)	22.9% (22.3%–23.6%)	1,471 (1,383–1,560)	6.3% (6.0%–6.7%)	
11–15 years	23,466 (23,160–23,771)	78.7% (78.2%–79.3%)	4,640 (4,486–4,794)	15.6% (15.1%–16.1%)	1,694 (1,600–1,789)	5.7% (5.4%–6.0%)	
16–20 years	24,310 (24,011–24,610)	81.0% (80.5%–81.5%)	4,371 (4,221–4,520)	14.6% (14.1%–15.0%)	1,325 (1,241–1,409)	4.4% (4.1%–4.7%)	

Race/ethnicity							
White	32,987 (32,656–33,317)	77.2% (76.7%–77.6%)	7,381 (7,193–7,569)	17.3% (16.8%–17.7%)	2,376 (2,264–2,487)	5.6% (5.3%–5.8%)	*p* < 0.004
Black	4,174 (4,027–4,320)	73.3% (71.9%–74.7%)	1,013 (939–1,088)	17.8% (16.6%–19.0%)	503 (451–556)	8.8% (8.0%–9.8%)	
Hispanic	21,928 (21,644–22,212)	75.8% (75.2%–76.3%)	5,458 (5,296–5,619)	18.9% (18.3%–19.4%)	1,558 (1,468–1,648)	5.4% (5.1%–5.7%)	
Others	9,302 (9,092–9,512)	74.1% (73.2%–75.0%)	2,375 (2,264–2,486)	18.9% (18.1%–19.7%)	880 (812–949)	7.0% (6.5%–7.6%)	

Median household income for the patient ZIP code							
1st quartile	18,020 (17,747–18,293)	72.6% (71.9%–73.2%)	5,358 (5,195–5,522)	21.6% (21.0%–22.2%)	1,456 (1,367–1,545)	5.9% (5.5%–6.2%)	*p* < 0.004
2nd quartile	15,635 (15,371–15,898)	73.9% (73.2%–74.6%)	4,268 (4,120–4,415)	20.2% (19.5%–20.8%)	1,251 (1,169–1,333)	5.9% (5.5%–6.3%)	
3rd quartile	16,698 (16,430–16,966)	78.4% (77.7%–79.0%)	3,300 (3,171–3,430)	15.5% (14.9%–16.1%)	1,299 (1,217–1,382)	6.1% (5.7%–6.5%)	
4th quartile	16,733 (16,471–16,994)	80.4% (79.7%–81.0%)	2,872 (2,752–2,992)	13.8% (13.3%–14.4%)	1,213 (1,133–1,293)	5.8% (5.5%–6.2%)	

Insurance							
Government	26,727 (26,413–27,041)	73.4% (72.8%–73.9%)	7,385 (7,195–7,575)	20.3% (19.8%–20.8%)	2,310 (2,200–2,420)	6.3% (6.1%–6.6%)	
Private	34,229 (33,894–34,564)	78.0% (77.5%–78.5%)	7,196 (7,009–7,383)	16.4% (16.0%–16.8%)	2,458 (2,344–2,571)	5.6% (5.4%–5.9%)	
Others	7,435 (7,243–7,626)	77.2% (76.2%–78.2%)	1,646 (1,551–1,741)	17.1% (16.2%–18.0%)	550 (495–605)	5.7% (5.2%–6.3%)	

Urban vs. rural							
Urban	59,492 (59,197–59,786)	77.7% (77.4%–78.1%)	12,495 (12,262–12,728)	16.3% (16.0%–16.6%)	4,556 (4,405–4,707)	6.0% (5.8%–6.2%)	*p* < 0.008
Rural	8,899 (8,720–9,078)	66.4% (65.5%–67.4%)	3,732 (3,603–3,861)	27.9% (26.9%–28.8%)	761 (697–826)	5.7% (5.2%–6.2%)	

Hospital type							
Children's hospital	12,484 (12,378–12,590)	84.8% (84.1%–85.5%)	852 (784–919)	5.8% (5.3%–6.3%)	1,388 (1,303–1,472)	9.4% (8.9%–10.0%)	*p* < 0.001
Non-children's hospital	55,906 (55,632–56,181)	74.3% (74.0%–74.7%)	15,375 (15,123–15,627)	20.4% (20.1%–20.8%)	3,930 (3,790–4,070)	5.2% (5.0%–5.4%)	

Complicated vs. noncomplicated appendicitis							
Complicated appendicitis	19,293 (19,012–19,575)	66.2% (65.6%–66.8%)	5,965 (5,791–6,138)	20.5% (19.9%–21.0%)	3,883 (3,742–4,024)	13.3% (12.9%–13.8%)	*p* < 0.0001
Noncomplicated appendicitis	49,098 (48,753–49,442)	80.8% (80.4%–81.1%)	10,262 (10,045–10,479)	16.9% (16.5%–17.2%)	1,435 (1,347–1,522)	2.4% (2.2%–2.5%)	

## Data Availability

Data from the Kid's Inpatient Database are used to support the study.

## Abbreviations

HCUP: Healthcare Cost and Utilization Project
KID: Kids' Inpatient Database
MHI: Median Household Income.
